# Large-scale genomic-wide CRISPR screening revealed PRC1 as a tumor essential candidate in clear cell renal cell carcinoma

**DOI:** 10.7150/ijms.107691

**Published:** 2025-03-03

**Authors:** Baochao Li, Yongsheng Pan, Jiajin Wu, Chenkui Miao, Zengjun Wang

**Affiliations:** Department of Urology, First Affiliated Hospital of Nanjing Medical University, No. 300, Guangzhou Street, Nanjing, Jiangsu Province 210029, China.

**Keywords:** clear cell renal cell carcinoma, PRC1, genome-wide CRISPR screening, bioinformation analysis, cancer progression

## Abstract

**Background**: Clear cell renal cell carcinoma (ccRCC) is a prevalent and aggressive subtype of kidney cancer, often associated with metastasis and recurrence. Identifying key genes involved in ccRCC progression is critical for improving treatment strategies and patient outcomes.

**Methods**: We performed a large-scale genome-wide CRISPR screening to identify genes crucial to ccRCC progression using the DepMap database. For discovery and validation, we integrated multi-omics data from The Cancer Genome Atlas (TCGA), GEO, and the NJMU-ccRCC clinical cohort. Bioinformatics analyses, including differential expression, pathway enrichment, and protein-protein interaction network analysis, were conducted to elucidate the biological functions. To validate our findings, we employed immunohistochemistry, qRT-PCR, and various cellular assays to investigate the role of PRC1 in ccRCC.

**Results**: CRISPR screening identified PRC1 as a key gene significantly overexpressed in ccRCC tissues from the DepMap database. Elevated PRC1 expression was associated with poor overall survival, disease-specific survival, and progression-free interval. Silencing PRC1 in ccRCC cell lines inhibited cell proliferation, migration, and colony formation. Functional enrichment analyses revealed that PRC1 is involved in essential processes such as cell cycle regulation, mitosis, and cytokinesis. Additionally, PRC1 expression was correlated with the activation of the Wnt/β-catenin pathway, suggesting that PRC1 plays a pivotal role in tumor progression.

**Conclusion**: PRC1 emerges as a promising biomarker and therapeutic target for ccRCC. Elevated PRC1 expression is associated with poor prognosis, and its inhibition suppresses ccRCC cell proliferation and migration. Our findings underscore the crucial role of PRC1 in ccRCC progression and highlight the need for further investigation into its molecular mechanisms and therapeutic potential.

## Introduction

Renal cancer is one of the common malignant tumors of the urinary system. Clear cell renal cell carcinoma (ccRCC) is the most prevalent subtype, characterized by its distinct histological features and genetic alterations^1^. Early-stage ccRCC typically has a favorable prognosis, primarily managed through surgical resection. However, approximately 30% of patients are diagnosed with metastatic disease, and 20%-40% of those undergoing surgery experience recurrence, leading to poorer outcomes^2^. Recent advances in genomics have highlighted numerous genes with abnormal expression in ccRCC, implicating their roles in tumorigenesis and progression. While these genetic factors are associated with the malignancy of ccRCC, their precise molecular mechanisms remain under investigation. Consequently, identifying novel biomarkers and elucidating their regulatory roles in ccRCC is crucial for advancing our understanding and treatment of this disease^3^, ^4^.

CRISPR/Cas9 is a powerful genome editing tool^5^, considered a revolutionary technology in the field of genetic research^6^. By artificially designing these two types of RNAs, it is possible to create sgRNAs (short guide RNAs) that direct Cas9 to make specific cuts in DNA^7^, ^8^. Based on this, CRISPR/Cas9 sgRNA libraries can be constructed targeting the whole genome^9^, allowing rapid creation of loss-of-function mutations and obtaining the desired phenotype for research^10^. In this project, we applied the CRISPR/Cas9 library knockout screening technology to the study of renal cancer driver genes, which provided a new direction for further research on the mechanism of key driver genes in renal cancer^11^. Combining CRISPR screening and multi-omics sequencing data^12^, as well as high-throughput sequencing data of renal cancer tissues from our group, we identified the PRC1 gene, which is significantly overexpressed in renal cancer and plays an important role in malignant progression^13^-^17^.

Microtubules (MT) play a crucial role in mitosis, cell proliferation, cycle regulation, and signal transduction, with changes in MT-associated proteins (MAPs) often linked to poor cancer prognosis^18^. PRC1 is essential in cytokinesis, localizing to the spindle midzone during mitosis, binding and bundling MTs, and recruiting other spindle proteins, thereby influencing MAP functions^17^, ^19^. Dysregulation of PRC1 disrupts MAP distribution, causing cytokinesis defects, chromosomal aneuploidy, instability, and cancer progression^20^, ^21^. PRC1 dysregulation is reported in various tumors, including hepatocellular carcinoma, breast cancer, and gastric cancer, correlating with high tumor grade, aggressiveness, and poor prognosis^22^-^24^. It also impacts tumor cell proliferation, apoptosis, and cycle regulation, with PRC1's role in Wnt/β-catenin signaling and the p53/PRC1/EGFR pathway being significant in cancer progression^25^. Despite its known effects in these cancers, PRC1's role in renal cell carcinoma (ccRCC) remains unexplored. In this research, we performed comprehensive bioinformation research revealed PRC1 was the essential gene in ccRCC progression. Our study showed that PRC1 was significantly overexpressed in renal cell carcinoma, and knockdown of PRC1 inhibited ccRCC cell proliferation, migration, both *in vitro* and *in vivo*, but the specific mechanism is not clear.

## Materials and methods

### Data acquisition and processing

Approved by the Ethics Committee of the First Affiliated Hospital of Nanjing Medical University, this study collected tissue samples confirmed as clear cell renal cell carcinoma (ccRCC). Cancerous and adjacent normal tissues (>3 cm from the tumor) were used as controls. Diagnosis was confirmed via immunohistochemistry, and tissues were frozen in liquid nitrogen. No patients had prior tumor-specific treatments. Informed consent was obtained from all patients. The Cancer Dependency Map (https://depmap.org/portal/) is an online resource derived from a comprehensive multi-omics screening initiative^26^, ^27^. The CRISPR Essentiality Scores (CERES) was employed to compute dependency scores across genes, pinpointing those vital for cellular proliferation and survival. Genes with a CERES score above zero suggest that gene disruption enhances cell line viability, whereas scores below zero imply inhibition of survival and proliferation. Genes with a CERES score less than -1 in over 75% of clear cell renal cell carcinoma (ccRCC) cell lines were considered as essential^28^.

Transcriptomic RNA-sequencing data and associated clinical information for ccRCC were procured from The Cancer Genome Atlas (TCGA) data portal (https://portal.gdc.cancer.gov/). The dataset included 538 ccRCC tissue samples and 72 normal tissue controls. Additionally, gene expression, prognostic, and therapeutic response analyses were conducted using cohorts GSE40435, GSE53000, GSE46699, GSE15641, GSE781, GSE6344, GSE53757, GSE66270, GSE66271, GSE105261, and GSE36895 from the GEO database (https://www.ncbi.nlm.nih.gov/geo/). RNA-Sequencing data from tumor and adjacent non-tumor tissues of ccRCC patients at Nanjing Medical University's Department of Urology (NJMU), along with data from the Clinical Proteomic Tumor Analysis Consortium (CPTAC) (https://proteomics.cancer.gov/programs/cptac), were integrated to identify key prognostic genes for ccRCC. For NJMU ccRCC clinical cohort, we collected surgical resection samples from patients diagnosed with clear cell renal cell carcinoma (ccRCC) between 2022 and 2024. Both tumor tissues and adjacent normal tissues were confirmed by at least two pathologists. For the NJMU ccRCC RNA sequencing cohort, we used 5 pairs of matched ccRCC tumor and adjacent normal kidney tissues, along with an additional ccRCC tumor sample. For the NJMU ccRCC PCR validation cohort, we used 32 pairs of matched samples for validation. Ethical approval was obtained from the Ethics Committee of Nanjing Medical University for all tissue donations used in this research, and informed consent was duly signed by all participating patients. Public databases utilized in this study are exempt from ethical review.

### Differential expression analysis

We utilized the “Limma” R package to identify differentially expressed genes (DEGs) between clear cell renal cell carcinoma (ccRCC) and normal kidney tissue within the TCGA and NJMU datasets. DEGs were defined as genes exhibiting an absolute log fold change (|logFC|) greater than 1 and a false discovery rate (FDR) below 0.05. Following this, the R pheatmap package was employed for the visualization of DEGs and key genes associated with ccRCC cell lines.

### Enrichment analysis and PPI network

Utilize the Metascape database (https://metascape.org), a complimentary gene annotation resource, to conduct an automated meta-analysis of expression data, facilitating the visualization of key biological pathways significantly associated with ccRCC and its dependent genes. STRING (https://string-db.org/), a comprehensive database of both established and hypothesized protein-protein interactions (PPI), was applied to illustrate the interaction network for DEGs. The critical genes within the PPI network are subjected to scrutiny using the Cytohubba plug-in (https://apps.cytoscape.org/apps/cytohubba) in Cytoscape. Metascape identifies enriched PPI clusters, leveraging the Molecular Complex Detection (MCODE) algorithm, which integrates vertex weighting, complex prediction, and post-processing techniques to pinpoint densely interconnected regions.

### Identifying prognostic biomarkers

The expression levels of key prognostic DEGs in ccRCC were obtained from the normalized expression matrix and integrated with patient data on survival time and status. Following this, the genes identified in the initial analysis were subjected to univariate Cox regression analysis to evaluate their association with overall survival (OS), disease-specific survival (DSS), and progression-free interval (PFI). Kaplan-Meier survival curves were constructed to assess the prognosis value of clear cell renal cell carcinoma (ccRCC) patients.

### Prognostic significance of PRC1 in ccRCC

Patients were stratified into two groups based on median PRC1 expression. Furthermore, univariate and multivariate Cox regression analyses were employed to investigate the potential of PRC1 expression and various clinical parameters as independent prognostic factors for ccRCC outcomes. For each variable, logistic regression analysis was conducted, and the odds ratios (ORs) along with 95% confidence intervals (CIs) were computed to identify the risk factors associated with ccRCC. To validate the prognostic significance of PRC1, receiver operating characteristic (ROC) analysis was performed. Following the insights gained from multivariate Cox proportional hazards analysis, a nomogram was developed for forecasting 1-, 3-, and 5-year overall survival, disease-specific survival, and recurrence probabilities for ccRCC patients using the 'rms' R package.

### Funtional enrichment and pathway annotation

We conducted Gene Set Enrichment Analysis (GSEA), Gene Ontology (GO) analysis, encompassing molecular function (MF), cellular components (CC), and biological processes (BP), as well as Kyoto Encyclopedia of Genes and Genomes (KEGG) pathway analysis.

### Immunohistochemical assays

Immunohistochemical assays were constructed utilizing tumor and adjacent non-tumor tissues from NJMU ccRCC patients. Immunohistochemical analysis was conducted using PRC1 primary antibody (1:200, Abcam, USA). This metric was utilized to assess the differential expression of the PRC1 protein between tumor tissues and adjacent non-tumor tissues.

### Real-time quantitative polymerase chain reaction (qRT-PCR)

Total RNA was extracted using TRIzol reagent (Thermo Fisher Scientific). After determination of the RNA concentration and purity, cDNA was synthesized using the HiScript® III All-in-one RT SuperMix Perfect (Vazyme Biotech Co., Ltd.). RT-qPCR was conducted using the ChamQ Universal SYBR qPCR Master Mix (Vazyme Biotech Co., Ltd.) by LightCycler® 96 Instrument (Roche. lifescience). The expression level was quantized by 2^-ΔΔCt^ mode. GAPDH was regarded as the reference gene for quantitative analysis. The primer sequences for RT-qPCR are listed in **Table S1**.

### CCK-8 assay

CCK-8 assay was used to measure cell proliferation. 786-O and Caki-1 cell lines, transfected with NC (negative control) and siRNA-PRC1, were seeded in 96-well plates (1*10^3^ cells/well) in quadruplicate and cultured for 24, 48, 72 and 96h. Two hours before absorbance measuring, a CCK-8 solution was added. The absorbance was measured at 450 nm with a microplate reader after incubation at 37°C.

### 5-Ethynyl-2′-deoxyuridine (EdU) assay

The cell proliferation assay was conducted using the BeyoClick™ EDU-488 kit (Beyotime, China) to detect EdU incorporation in Caki-1 and 786-O cells, following the manufacturer's instructions. Cells transfected with either NC or siR-PRC1 (1*10^5^ cells per well) were seeded onto 6-well plates. After 24 hours, the medium was supplemented with 10 mM EdU and incubated for 2 hours. Subsequently, the cells were fixed with 4% paraformaldehyde for 15 minutes. After fixation, the cells underwent three washes with a buffer containing 3% BSA, followed by permeabilization with 0.3% Triton X-100 in PBS for 15 minutes. After a final wash, the Click-iT reaction mixture was added, and the cells were incubated in the dark at room temperature for 30 minutes. This procedure labeled proliferating cells, which were then counterstained with DAPI to visualize nuclei. Fluorescence microscopy was employed to examine the cells.

### Colony formation assay

786-O and Caki-1 cells were seeded into a 6-well plate at a density of 1,000 cells per well. Following a 10-day incubation period, cells were fixed with 4% paraformaldehyde for 15 minutes. Subsequently, cells were stained with a 0.1% crystal violet solution for 30 minutes. The resulting colonies were imaged and quantified.

### Transwell assay

The migratory capacity of the cells was evaluated using the Transwell assay. Specifically, 786-O and Caki-1 cell lines were seeded into the upper chamber of the Transwell apparatus at a density of 1*10^5^ cells/well in 200 µL of serum-free medium. The lower chamber was filled with 500 µL of medium supplemented with 10% fetal bovine serum. After incubation for 24 hours, the cells that had migrated through the membrane were quantified utilizing a fluorescence microscope.

### Statistical analysis

Bioinformatic analyses were performed using R software (version 4.3.3). Data are presented as mean ± standard deviation. Student's t-test was used to determine the statistical significance of differences between two groups, while ANOVA was used for multiple group comparisons. Fisher's exact test, Chi-square test, Wilcoxon signed-rank test and logistic regression analysis were used to assess the association between PRC1 expression and clinicopathological characteristics. The diagnostic performance was evaluated using receiver operating characteristic (ROC) curve analysis. The impact of PRC1 expression on prognosis was assessed by Kaplan-Meier survival analysis and Cox regression modelling. Statistical analyses of immunohistochemistry (IHC) and quantitative real-time PCR (qRT-PCR) were performed using GraphPad Prism v9.0 (GraphPad Software, Inc., San Diego, CA, USA). A *P*-value < 0.05 was considered statistically significant.

## Results

### Identification of key genes in ccRCC functional genomics

**Figure 1:**
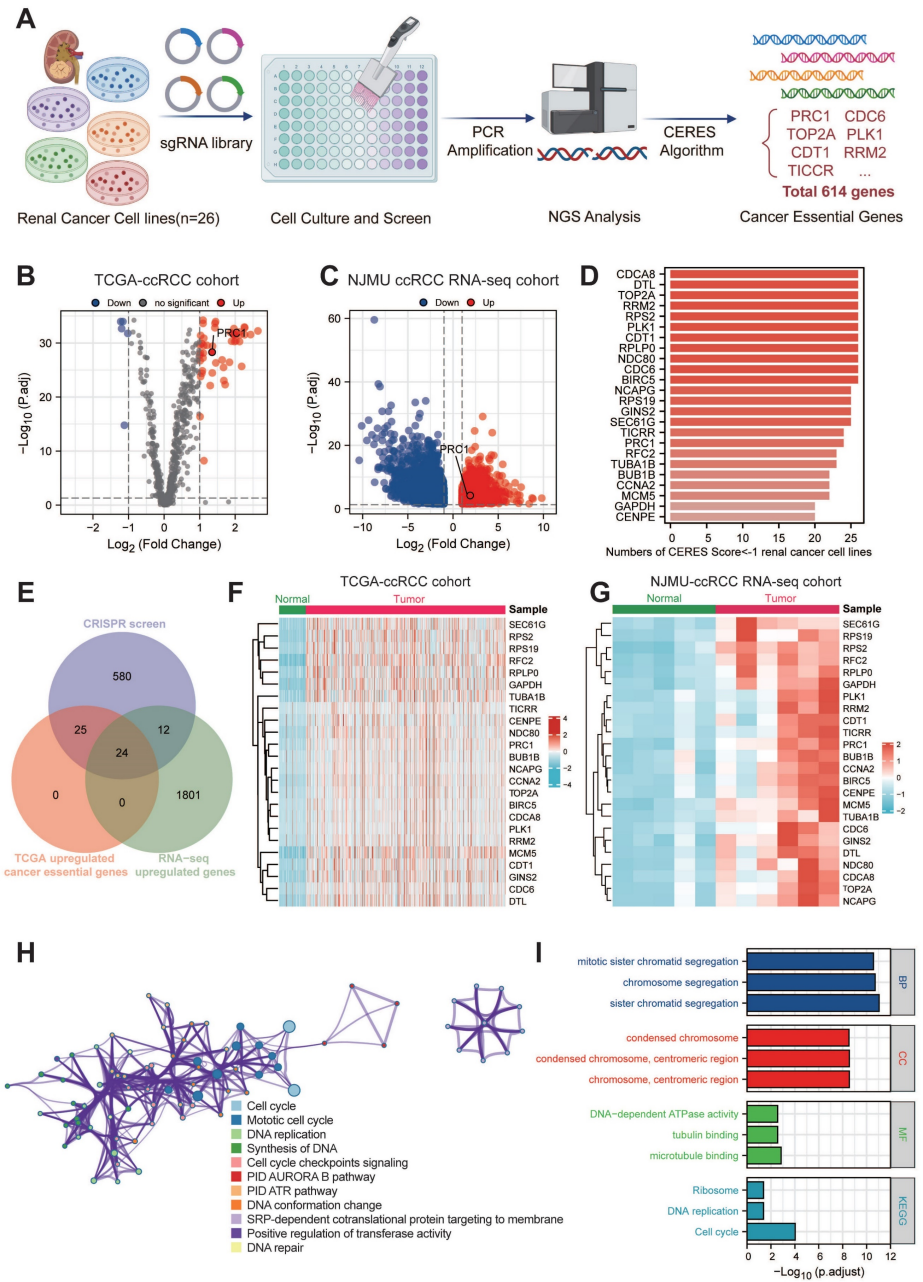
** Large-scale genomic-wide CRISPR screening identified key regulators in ccRCC.** (A). The schematic diagram of the overall genomic-wide CRISPR screening process of this study design. (B). The volcano plot illustrates the differential expression of 614 essential genes in the TCGA-KIRC cohort. (C). The volcano plot illustrates the differential expression of 614 essential genes in the NJMU-ccRCC cohort. (D). Bar plot showed the numbers of CERES score < -1 renal cancer cell lines. (E). Venn diagram showed the intersection of CRISPR essential genes, TCGA up-regulated genes and NJMU RNA-seq up-regulated genes. (F). Heatmaps illustrated the expression patterns of these key regulators in TCGA-ccRCC cohort. (G). Heatmaps illustrated the expression patterns of these key regulators in NJMU ccRCC cohort. (H). Cytoscape visualized the intrinsic connections and interaction patterns among these genes. (I). Functional enrichment and pathway annotation of these essential regulators.

The overall genomic-wide CRISPR screening process of this study was illustrated in the **Figure 1A**. We retrieved CERES dependency scores for nine ccRCC cell lines from the DepMap portal and categorized genes as candidates if they had a CERES score below -1 in more than 75% of the ccRCC cell lines evaluated. This process identified 641 candidate genes critical for the survival of 26 ccRCC cell lines. Subsequently, our analysis focused on distinguishing these 641 candidate genes from aberrant gene expression in ccRCC. Through DEGs analysis, we identified 49 candidate genes that were significantly upregulated in ccRCC tissues (n=538) compared to normal tissues (n=72) using data from the TCGA database (**Figure 1B**). In addition, in the NJMU database, we also found that 36 of these 49 genes exhibited high expression levels in ccRCC tissues (**Figure 1C**). Taken together, we identified 24 essential genes as potent tumor-dependent genes for further research (**Figure 1D** and **Table S2**).

To further investigate the expression patterns and biological functions of 24 key genes in ccRCC, we mapped the heat maps of these key oncogenes using the TCGA and NJMU databases, highlighting the differences in expression between tumor tissues and neighboring tissues (**Figure 1F-G**). Notably, a subset of these genes showed strong correlation with each other. We then entered these 24 genes into the Metscape tool for pathway enrichment analysis and found significant enrichment in both cell cycle and mitotic cycle pathways (**Figure 1H-I**). These genes also showed high association with DNA synthesis and replication and microtubule binding. Further analysis by KEGG and GO revealed that these genes are involved in chromosome segregation, condensation of chromosomes and mitotic regions, and are closely associated with cell proliferation.

### PRC1 is an essential driving factor for ccRCC progression

**Figure 2:**
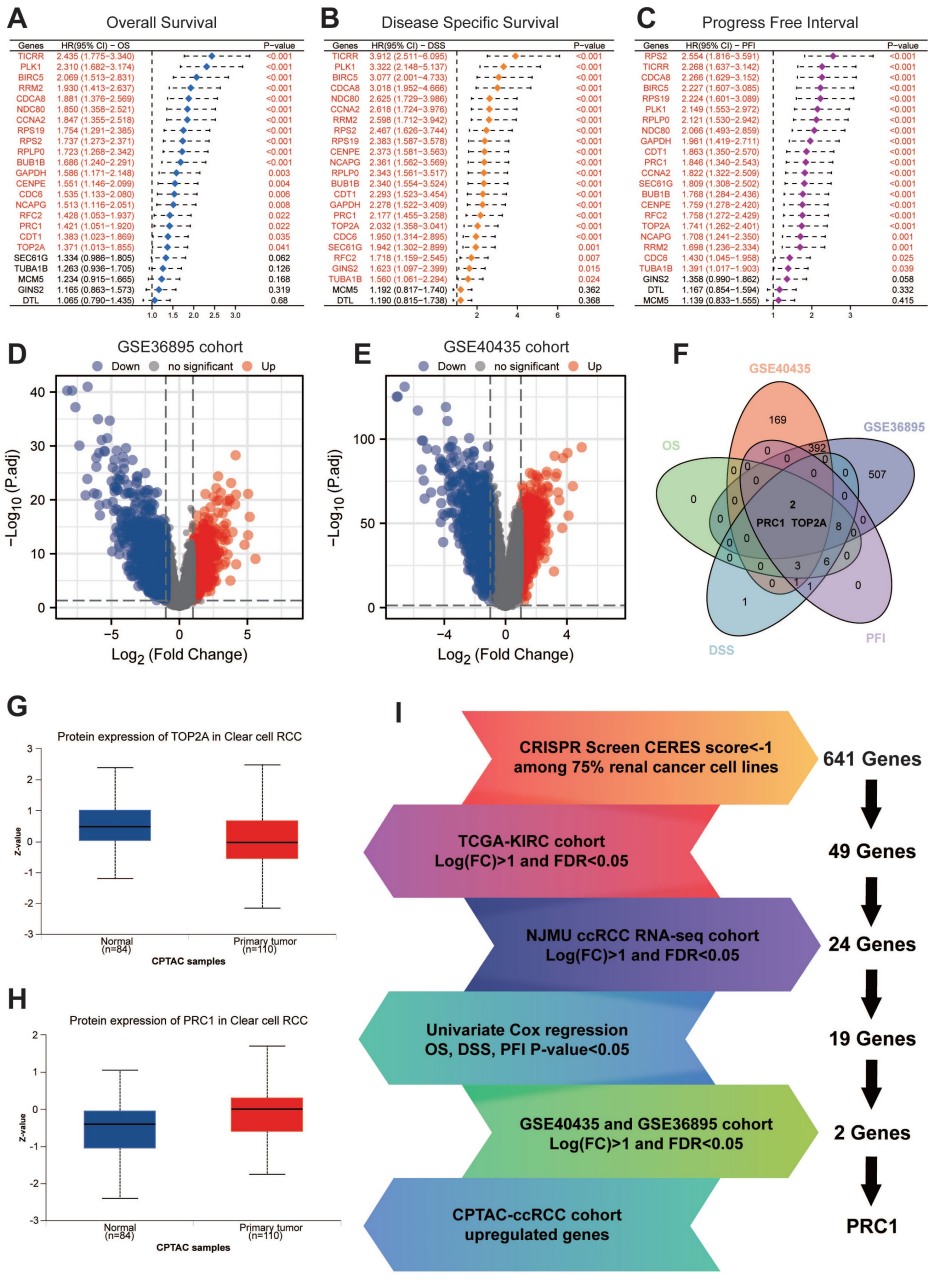
** Further validation and screening revealed PRC1 is an essential driving factor for ccRCC.** (A). Univariate Cox regression analysis revealed the association between the expression level of 24 essential genes and ccRCC patients' overall survival. (B). Univariate Cox regression analysis revealed the association between the expression level of 24 essential genes and ccRCC patients' disease specific survival. (C). Univariate Cox regression analysis revealed the association between the expression level of 24 essential genes and ccRCC patients' progress free interval. (D). The volcano plot illustrates the differential expression of genes in the GSE36895 cohort. (E). The volcano plot illustrates the differential expression of genes in the GSE40435 cohort. (F). Venn diagram showed the intersection of poor OS, DSS, PFI, up-regulated genes in GSE40435 and GSE36895 cohort. (G). The protein expression level of TOP2A in CPTAC cohort. (H). The protein expression level of PRC1 in CPTAC cohort. (I). The summary flowchart of the entire large-scale screening process.

We subsequent applied univariate cox regression analysis assessed the impact of 24 genes on ccRCC patients' OS, DSS and PFI. The results showed that 19 of these genes were significantly associated with OS, DSS and PFI (**Figure 2A-C**). Notably, TOP2A and PRC1 were these 19 key prognostic genes, which were also identified as differential genes in the GSE40435 and GSE36895 datasets comparing paraneoplastic and tumor tissues (**Figure 2D-F**). Ultimately, PRC1 was the only gene in the CPTAC database that exhibited significantly higher protein levels in tumor tissues compared to paraneoplastic tissues (**Figure 2G-I**).

### PRC1 was up-regulated in ccRCC tumor samples

**Figure 3:**
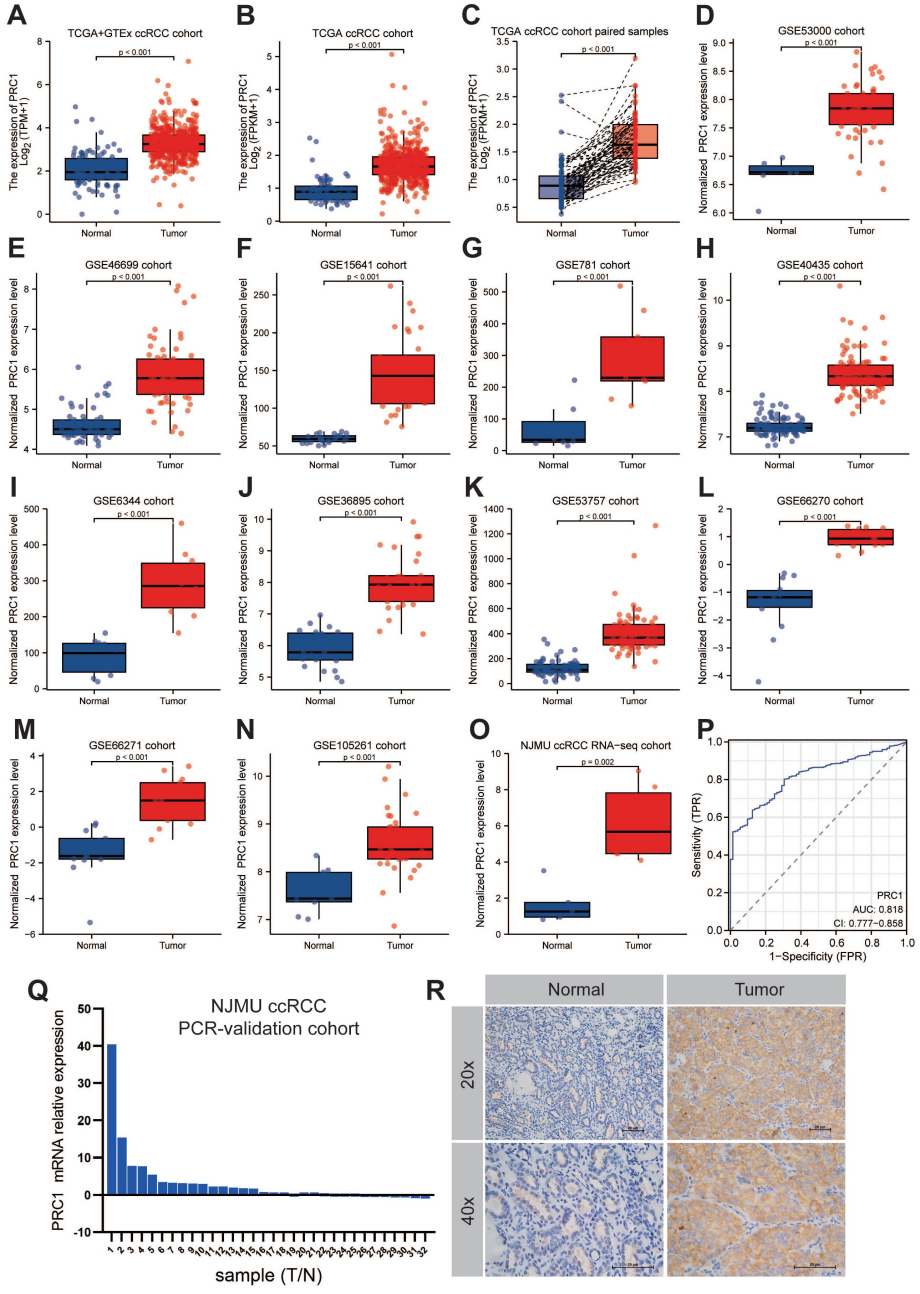
** The expression patterns of PRC1 in cinical ccRCC and normal kidney samples.** (A-O). The expression level of PRC1 in TCGA+GTEx cohort (A), TCGA-ccRCC cohort (B), TCGA-ccRCC paired samples (C), GSE53000 cohort (D), GSE46699 cohort (E), GSE15641 cohort (F), GSE781 cohort (G), GSE40435 cohort (H), GSE6344 cohort (I), GSE36895 cohort (J), GSE53757 cohort (K), GSE66270 cohort (L), GSE66271 cohort (M), GSE105281 cohort (N) and NJMU ccRCC RNA-seq cohort (O). (P). The diagnostic accuracy of PRC1 expression for ccRCC in TCGA ccRCC cohort (AUC = 0.818, 95% CI: 0.777-0.858). (Q). The fold change of PRC1 between ccRCC tumor and adjacent normal kidney tissues in NJMU-ccRCC cohort. (R). Immunohistochemical experiments confirmed the high expression of PRC1 in tumor tissues of ccRCC.

Next, we further analyzed the expression patterns of PRC1 in ccRCC and adjacent normal kidney tissues among the NJMU ccRCC clinical cohort, TCGA and Genotypic Tissue Expression (GTEx) databases, as well as 11 GEO datasets, including GSE53000, GSE46699, GSE15641, GSE781, GSE40435, GSE6344, GSE36895, GSE53757, GSE66270, GSE66271 and GSE105261 cohort. Consistent with our expected results, PRC1 expression level was higher in tumor tissues compared with adjacent normal kidney tissues, highlighting the outstanding diagnostic and prognostic performance of PRC1 in ccRCC progression (**Figure 3A-O**). The expression levels of PRC1 in the TCGA database and the corresponding clinicopathological information are shown in **Table 1**. Furthermore, Quantitative real-time PCR experiment of clinical samples revealed significantly higher PRC1 expression levels in ccRCC tumor samples compared with adjacent normal kidney tissues (**Figure 3P**). We assessed the diagnostic accuracy of PRC1 expression for ccRCC, and the ROC curve indicated an AUC value of 0.818 (95% CI: 0.777-0.858), suggesting that PRC1 can serve as a reliable biomarker for the diagnosis of ccRCC (**Figure 3P**). Additionally, immunohistochemical analysis of kidney cancer and adjacent tissues from NJMU ccRCC cohort indicated absent PRC1 expression in normal controls, while moderate expression was observed in ccRCC tissues, further underscoring PRC1's importance in renal cancer pathogenesis (**Figure 3Q**).

### Up-regulated expression PRC1 predicted worse survival outcomes

**Figure 4:**
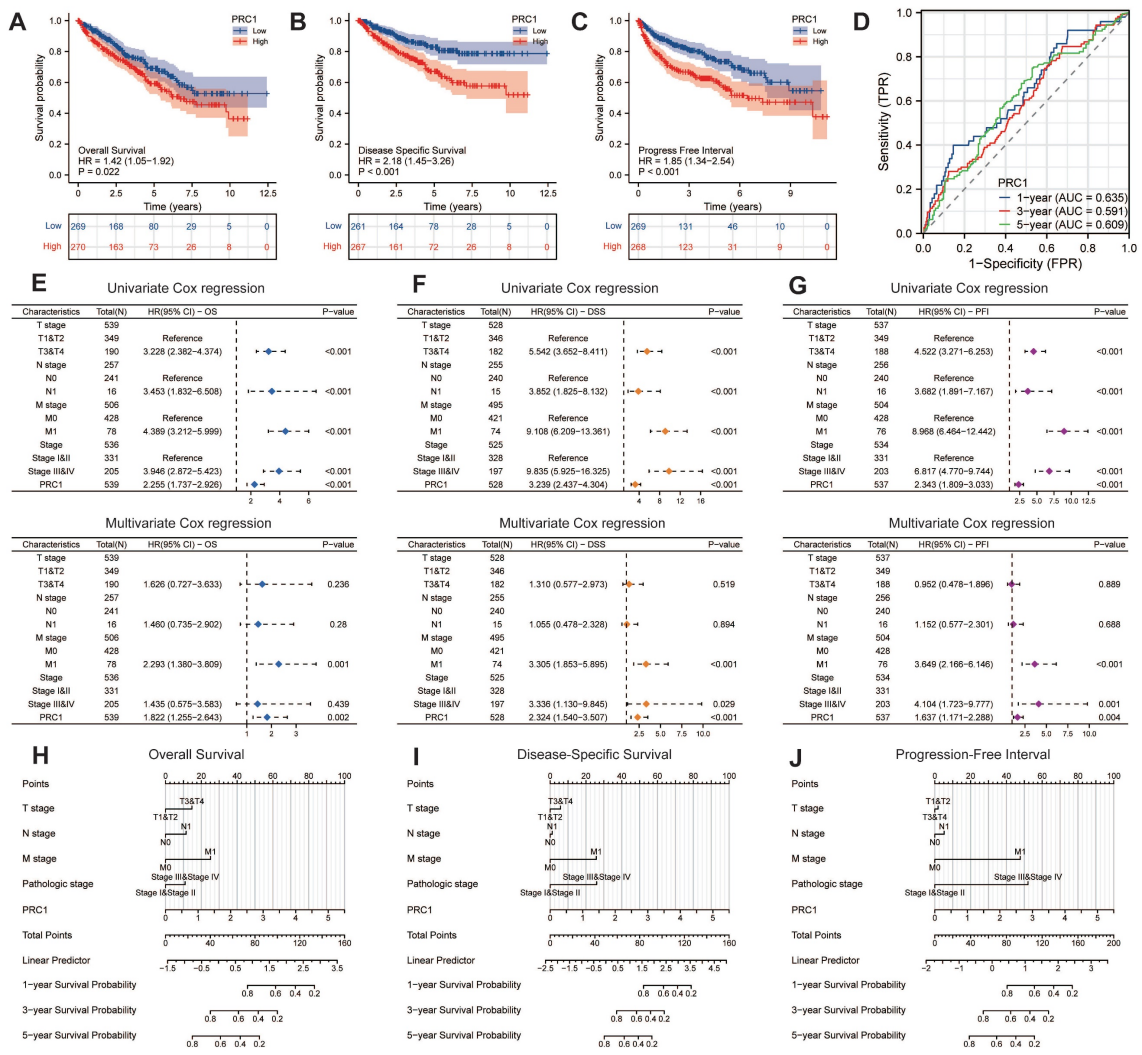
** Prognostic assessment of PRC1 in ccRCC patients.** (A). The Kaplan-Meier survival curve demonstrated the relationship between PRC1 expression and overall survival in ccRCC patients. (B). The Kaplan-Meier survival curve demonstrated the relationship between PRC1 expression and disease specific survival in ccRCC patients. (C). The Kaplan-Meier survival curve demonstrated the relationship between PRC1 expression and progress free interval in ccRCC patients. (D). Time-dependent ROC curve demonstrated the diagnostic efficacy of PRC1 expression in relation to 1-year, 3-year, and 5-year overall survival of ccRCC patients. (E-G). Univariate Cox regression and multivariate Cox regression analysis revealed up-regulated PRC1 expression was an independent risk factor for overall survival (E), disease specific survival (F) and progress free interval (G) in ccRCC patients. (H-J). Nomogram showed the prediction efficacy of PRC1 expression in relation to 1-year, 3-year, and 5-year for overall survival (H), disease specific survival (I) and progress free interval (J) of ccRCC patients.

We further examined the correlation between PRC1 expression and the survival prognosis of ccRCC patients. Patients from the TCGA database were stratified into high-risk and low-risk cohorts based on the median relative expression levels of PRC1. Survival analyses revealed that patients in the low-expression cohort exhibited significantly improved OS, DSS, and PFI compared to those in the high-expression cohort. Elevated PRC1 expression correlated with adverse prognostic outcomes, achieving a prediction accuracy (AUC) of 0.635 in 1-year, 0.591 in 3-year and 0.609 in 5-year (**Figure 4A-D**). Therefore, PRC1 is of considerable and reliable clinical biomarker. Additionally, integrating various independent prognostic indicators, we developed three nomogram prognostic charts for ccRCC patients, each substantiated by p-values less than 0.05 in multivariate Cox regression analyses for OS, DSS, and PFI. These charts predict the survival probabilities of ccRCC patients at various postoperative intervals based on the cumulative points derived from individual prognostic parameters (**Figure 4E-G**). Specifically, they offer precise predictions of mortality, cancer-specific mortality, and recurrence risk at 1-, 3-, and 5-years post-surgery (**Figure 4H-J**). Our findings underscore the significance of PRC1 expression as an autonomous prognostic marker that, when integrated with conventional clinical parameters, forecasts the prognostic outlook for ccRCC patients effectively.

### The potential biological function of PRC1 in ccRCC

**Figure 5:**
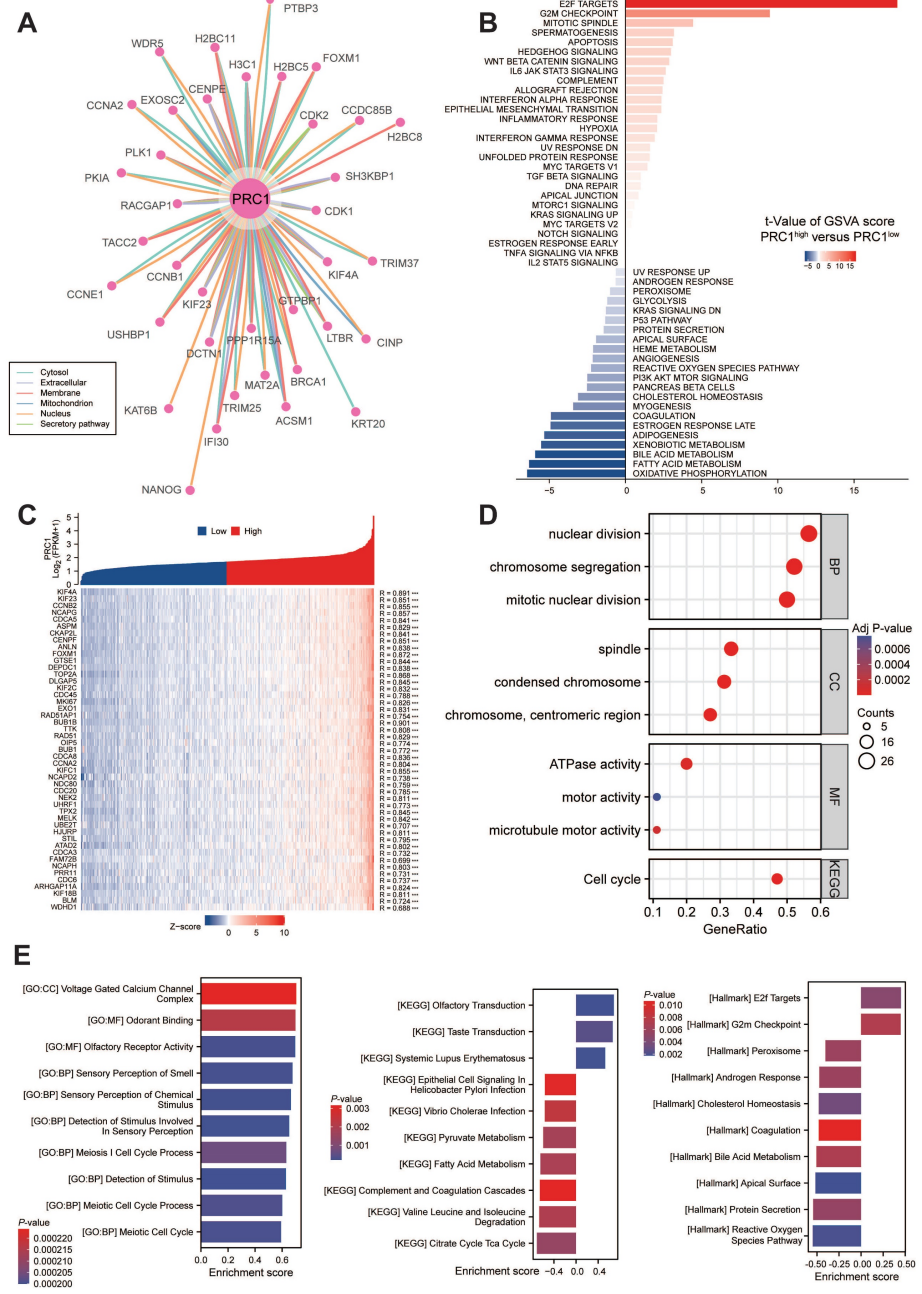
** Functional enrichment and pathway annotation of PRC1 in ccRCC patients.** (A). ComPPI database for constructing a cellular compartment-specific protein-protein interaction network of PRC1. (B) GSVA analysis illustrated that PRC1 participated in Hallmark cancer related pathways. (C). The correlation analysis between PRC1 expression and positive-correlated expression genes. (D). GO and KEGG functional enrichment of PRC1 among TCGA-ccRCC cohort. (E). GSEA results of PRC1 functional enrichment analysis using Hallmark, GO and KEGG gene sets.

The PPI network revealed interactions involving PRC1 and its co-localization with interacting proteins. To further elucidate PRC1's role, we conducted an analysis of genes co-expressed with PRC1 from TCGA dataset. We performed GSVA analysis to investigate the underlying biological functions. The results showed that the top 50 genes related to PRC1 were all positively correlated with its expression in KIRC, as demonstrated in the accompanying plots (**Figure 5A**). High expression levels of PRC1 were associated with the activation of biological processes such as E2F targets, G2/M checkpoint, and mitotic spindle assembly, compared to samples with lower expression levels. These processes are crucial for cell cycle progression and mitosis, suggesting that elevated PRC1 expression may enhance cell proliferation and division. Additionally, high PRC1 expression was examined in relation to processes like apoptosis, Hedgehog signaling, and Wnt/β-catenin signaling (**Figure 5B-C**).

The findings suggest that increased PRC1 expression may suppress cell death and differentiation. In terms of BP, PRC1 exhibited a strong correlation with cell division and chromosome separation. In the CC category, it showed a significant correlation with chromosome condensation and spindle assembly. These observations collectively indicate a pivotal role for PRC1 in cell cycle regulation (**Figure 5D**). Correspondingly, we performed a correlation analysis between the expression levels of PRC1 mRNA and homologous recombination repair genes. The results indicated a significant positive correlation between the expression of PRC1 and these genes. This finding further supports the involvement of the homologous recombination repair pathway in the biological mechanism by which PRC1 regulates the progression of ccRCC tumors (**Figure S1A**). Furthermore, we performed GSEA enrichment analysis on PRC1, and the results indicated that PRC1 is highly associated with pathways such as GO:CC Voltage gated calcium channel complex, and KEGG olfactory receptor activity. PRC1 primarily promotes the progression of ccRCC tumors through Hallmark E2F targets and G2M checkpoint pathways (**Figure 5E**). Ultimately, we also analyzed the correlation between PRC1 expression levels and the infiltration of immune cells in the tumor microenvironment. The results indicated that there was no significant correlation between PRC1 expression and the Immune Score, ESTIMATE Score or Stromal Score (**Figure S2A**). Subsequently, we used algorithms such as Cibersort and ssGSEA algorithms to assess the infiltration levels of specific immune cells. We found that PRC1 expression was significantly positively correlated with the abundance of T helper cells, T central memory cells, T effect memory cells, and other cells, while it showed a significant negative correlation with DC cells and others (**Figure S2B-C**).

### Overexpression of PRC1 in ccRCC enhances tumor cell proliferation and migration

**Figure 6:**
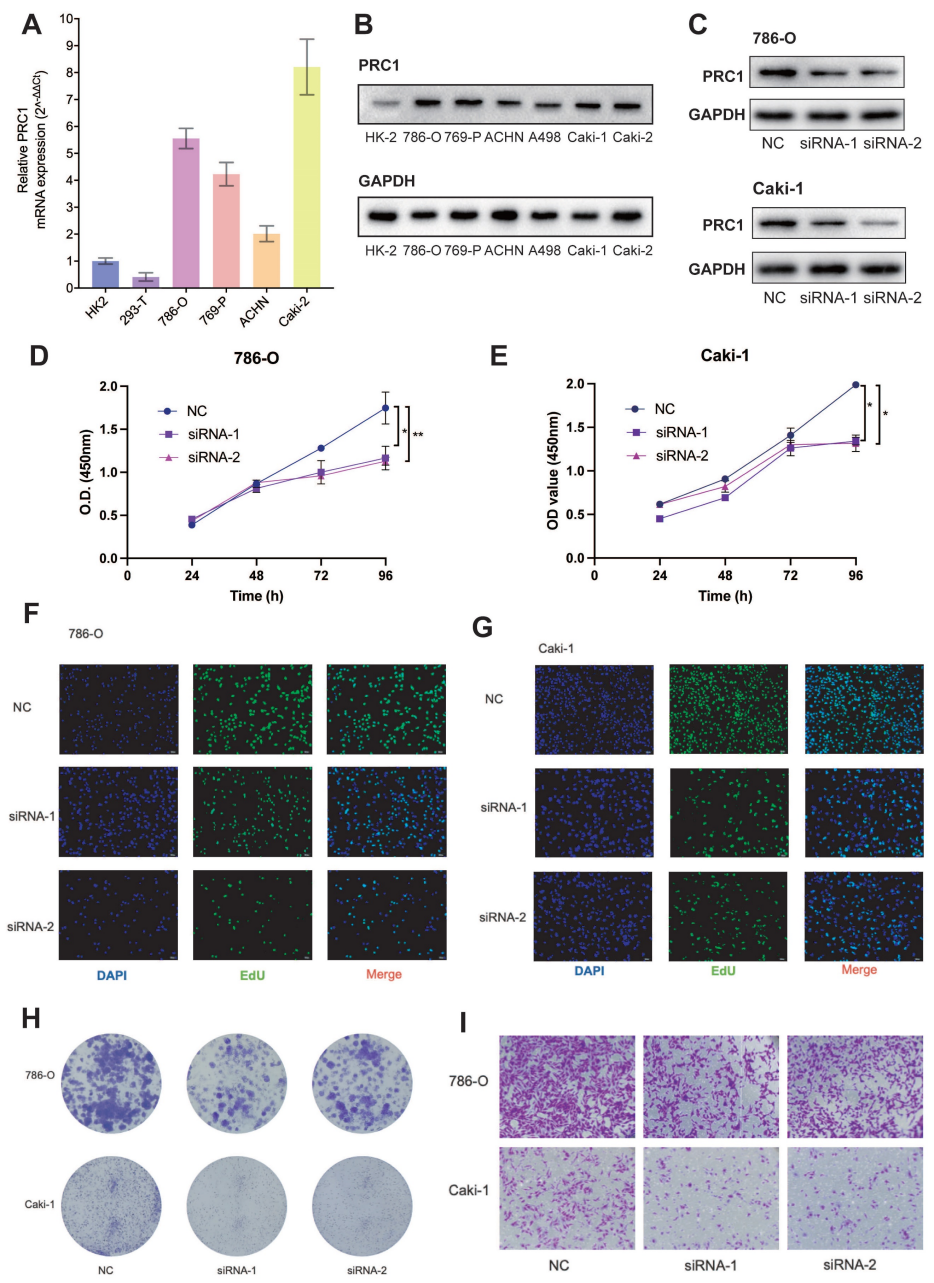
** Knockdown PRC1 significantly suppressed proliferation and migration of ccRCC cells.** (A). qRT-PCR experiments to validate PRC1 was up-regulated in ccRCC cancer cell lines. (B). Western bolt assays PRC1 was up-regulated in ccRCC cancer cell lines. (C). Western bolt assays PRC1confirm the performance of siRNA targeting PRC1 among 786-O and Caki-1 ccRCC cells. (D-E). CCK-8 assay results indicated that PRC1 knockdown decreased cell proliferation among 786-O (D) and Caki-1 (E) ccRCC cells. (F-G). EdU assay results indicated that PRC1 knockdown decreased cell proliferation among 786-O (F) and Caki-1 (G) ccRCC cells. (H). Colony-formation efficiency of knockdown PRC1 in 786-O and Caki-1 cells. (I). Transwell migration assay of knockdown PRC1 and control group.

Next, we investigated the expression levels of PRC1 in ccRCC cancer cell lines and normal renal tubular epithelial cell line HK-2. Quantitative real-time PCR analysis of four renal cancer cell lines revealed significantly higher PRC1 levels compared to HK-2 and 293-T cell lines (**Figure 6A**). To further determine the biological function of PRC1, we transfected 786-O and Caki-1 ccRCC cell lines with PRC1 small interfering RNA (siRNA), or control siRNA. PRC1 protein expression levels were measured by western blot assay to confirm the performance of siRNA (**Figure 6C**). Subsequently, we examined the impact of PRC1 on the proliferation and migration of ccRCC using assays such as CCK-8, EdU, colony formation, and Transwell migration. PRC1 expression in 786-O and Caki-1 cells was downregulated through siRNA-mediated knockdown. The outcomes of the CCK-8, EdU, and colony formation assays indicated that PRC1 silencing markedly inhibited the proliferative and colony-forming capacities of both 786-O and Caki-1 cells (**Figure 6D-H**). Additionally, the Transwell migration assay demonstrated that reduced PRC1 expression significantly diminished the migratory potential of ccRCC cells (**Figure 6I**). Taken together, the above results confirmed that PRC1 played a role of a positive regulator of tumor proliferation and migration in ccRCC.

To further validate our hypothesis that targeting PRC1 does not significantly affect the survival of normal renal cells, we performed knockdown PRC1 mRNA experiments in HK-2 cells and assessed their proliferation and migration abilities. The results indicated that silencing PRC1 had no significant effect on the proliferation or migration capacity of HK-2 cells (**Figure S4**). Overall, these findings further validate that PRC1 is a reliable biomarker for ccRCC treatment and a potential target for therapeutic intervention.

## Discussion

In this study, we employed genomic-wide CRISPR screening and multi-omics techniques to elucidate the upregulation of the PRC1 gene in renal cell carcinoma (RCC), highlighting its pivotal role in the malignant progression of the disease. We observed that PRC1 expression levels were significantly elevated in ccRCC tissues compared to normal renal tissues, consistent with the dysregulated expression of PRC1 in various tumor types^29^, ^30^. Furthermore, there was a strong correlation between high PRC1 expression and poor prognosis in ccRCC patients, suggesting that PRC1 is a viable biomarker and therapeutic target.

The primary indication that PRC1 serves as a prognostic marker for ccRCC emerges from several key observations. Firstly, PRC1 exhibits elevated expression in ccRCC relative to non-tumor tissues, a finding corroborated by data from TCGA, GEO, CPTAC, and the NJMU dataset. Moreover, the proliferation of 75% of RCC cell lines is contingent upon PRC1. Additionally, OS, PFI, and DSS are markedly poorer in patients with heightened intratumoral levels of PRC1. This relationship remains statistically significant in both univariate and multivariate analyses, signifying that PRC1's prognostic implications are independent of commonly used clinical prognostic factors, such as TNM stage and clinical stage.

Tumor progression and advanced metastasis involve multiple mechanisms, including the tumor microenvironment and pathways that promote tumor-associated angiogenesis^31^, ^32^. PRC1 safeguards cell division process by regulating microtubule binding and central spindle assembly^15^. Recent studies found that it might also play a role in tumorigenesis. Knockdown or knockout of PRC1 inhibited the growth, metastasis, recurrence, and/or drug resistance of various cancers^23^, ^24^, ^33^, ^34^. The underlying mechanisms have not been fully understood. Chen *et al.*^35^ was the first to identify an abnormal expression of PRC1 in liver hepatocellular carcinoma through analysis of microarray data. Their research delved into the transcriptional regulators influencing PRC1 expression, specifically examining the PRC1 promoter region's binding sites. This study effectively elucidated the reciprocal enhancement between PRC1 expression and the Wnt/β-catenin signaling pathway. Our bioinformatics analysis revealed a significant association between PRC1 and the Wnt/β-catenin pathway in KIRC, indicating PRC1 involved in crucial biological functions such as cell cycle progression, mitosis, and potentially affecting cell proliferation and division.

Using IHC experiments, predominant PRC1 localization was observed within the cytoplasm and along the cellular membrane. Consistent with previous reports, PRC1 distribution is dynamic, intricately linked to the cell cycle and mitotic processes, and has established associations with the CDK16/CCNY complex^29^. Notably, CDK1, CDK2, and CDK6^36^ play critical roles in the spatiotemporal regulation of this localization, predominantly residing within the nuclear compartment throughout the cell cycle. In the study by Xu *et al.*, it was found that PRC1 can affect the cell cycle, and inhibiting the expression of PRC1 can reduce the proliferation and colony-forming ability of colon cancer cells, block more G2/M phase cells, and promote cell apoptosis^37^.

Our biogenic analysis further corroborates the close association between PRC1 and the PLK1 signaling pathway, aligning with findings from prior research on Ewing's sarcoma. Notably, elevated PRC1 expression in EWS suggests a heightened sensitivity to the PLK1 inhibitor volasertib^38^. PLK1 activation of PRC1 leads to the formation of a PRC1-PLK1 protein complex, crucial for relocating PRC1 to the central spindle, thereby initiating cell division. Conversely, PLK1 can also exert a negative regulatory effect on PRC1, inhibiting the premature establishment of an intermediate compartment before cell division^39^. Currently, PLK1 inhibitors are being evaluated in clinical trials across a range of cancers. The expression level of PRC1 may serve as a predictive biomarker for the anti-tumor effectiveness of PLK1 inhibitors, further underscoring the importance of PRC1 research in clear ccRCC.

In this study, we identified that PRC1 emerged as a potential biomarker for ccRCC, discovered through multi-omics, CRISPR screenings. PRC1 appears to enhance proliferation, colony formation, and migration in ccRCC cell lines. Additionally, bioinformatics analyses hinted at PRC1's cellular roles, protein interactions, and involvement in signaling pathways within ccRCC. Despite these insights, a significant limitation lies in the lack of experimental validation for the bioinformatics predictions. Our research is undertaking mechanistic studies to substantiate these results and further elucidate PRC1's function in ccRCC, hoping to uncover novel therapeutic approaches.

## Conclusion

Our genome-wide CRISPR screening identified PRC1 as a key gene in clear cell renal cell carcinoma. Overexpressed in ccRCC tissues, PRC1 correlates with poor survival outcomes. PRC1 knockdown reduces ccRCC cell proliferation, migration, and colony formation, implicating its role in tumor growth. Functional analyses reveal PRC1's involvement in cell cycle regulation and key signaling pathways. These findings suggest PRC1 is a promising prognostic biomarker and therapeutic target, warranting further research to improve ccRCC treatment strategies.

## Supplementary Material

Supplementary figures and tables.

## Figures and Tables

**Table 1:** Clinicopathological information of PRC1 expression level among TCGA ccRCC database.

Characteristics	Low expression of PRC1	High expression of PRC1	*P*-value
n	270	271	
Age, n (%)			0.966
<= 60	134 (24.8%)	135 (25%)	
> 60	136 (25.1%)	136 (25.1%)	
Gender, n (%)			0.674
Female	91 (16.8%)	96 (17.7%)	
Male	179 (33.1%)	175 (32.3%)	
Histologic grade, n (%)			0.718
G1	6 (1.1%)	8 (1.5%)	
G2	118 (22.1%)	118 (22.1%)	
G3	107 (20.1%)	100 (18.8%)	
G4	34 (6.4%)	42 (7.9%)	
Pathologic stage, n (%)			0.841
Stage I	139 (25.8%)	134 (24.9%)	
Stage II	31 (5.8%)	28 (5.2%)	
Stage III	62 (11.5%)	61 (11.3%)	
Stage IV	38 (7.1%)	45 (8.4%)	
Pathologic T stage, n (%)			0.359
T1	143 (26.4%)	136 (25.1%)	
T2	40 (7.4%)	31 (5.7%)	
T3	83 (15.3%)	97 (17.9%)	
T4	4 (0.7%)	7 (1.3%)	
Pathologic N stage, n (%)			0.155
N0	120 (46.5%)	122 (47.3%)	
N1	5 (1.9%)	11 (4.3%)	
Pathologic M stage, n (%)			0.183
M0	225 (44.3%)	204 (40.2%)	
M1	35 (6.9%)	44 (8.7%)	
