# Supplementary material for: Large-scale genomic-wide CRISPR screening revealed PRC1 as a tumor essential candidate in clear cell renal cell carcinoma
Source: Int J Med Sci. 2025 Mar 3;22(7):1658–71. doi: 10.7150/ijms.107691 (PMC11905274; doi:10.7150/ijms.107691)

Supplementary Materials
Supplementary Tables
Table S1. Primer information for qRT-PCR experiment.
Table S2. Detailed 24 tumour essential candidate genes.

Supplementary Figures
Figure S1. The correlation analysis between the expression levels of PRC1 mRNA and homologous recombination repair genes.
Figure S2. Correlation between PRC1 expression levels and the infiltration of immune cells in the tumor microenvironment.
Figure S3. Quantitative bar graphs of in vitro experiments.
Figure S4. Assessment of the proliferation and migration abilities after knockdown PRC1 in HK-2 cells

Supplementary Tables

Table S1. Primer information for qRT-PCR experiment.

Genes	Primer (5’-3’)
PRC1-F	5′-ACAGACAGAGACAGAGATG-3′
PRC1-R	5′- GCCGAATGCTACTATTGG-3′
GAPDH-F	5'-GAAGGTGAAGGTCGGAGTC-3'
GAPDH-R	5'-GAAGATGGTGATGGGATTTC-3'

Supplementary Table 2. Detailed 24 tumour essential candidate genes.

Genes
BIRC5
CDC6
MCM5
NDC80
CCNA2
RPLP0
CENPE
PRC1
CDT1
SEC61G
GAPDH
GINS2
PLK1
TUBA1B
RPS2
TICRR
RFC2
RRM2
TOP2A
RPS19
BUB1B
DTL
NCAPG
CDCA8

Supplementary Figures

Figure S1. The correlation analysis between the expression levels of PRC1 mRNA and homologous recombination repair genes.

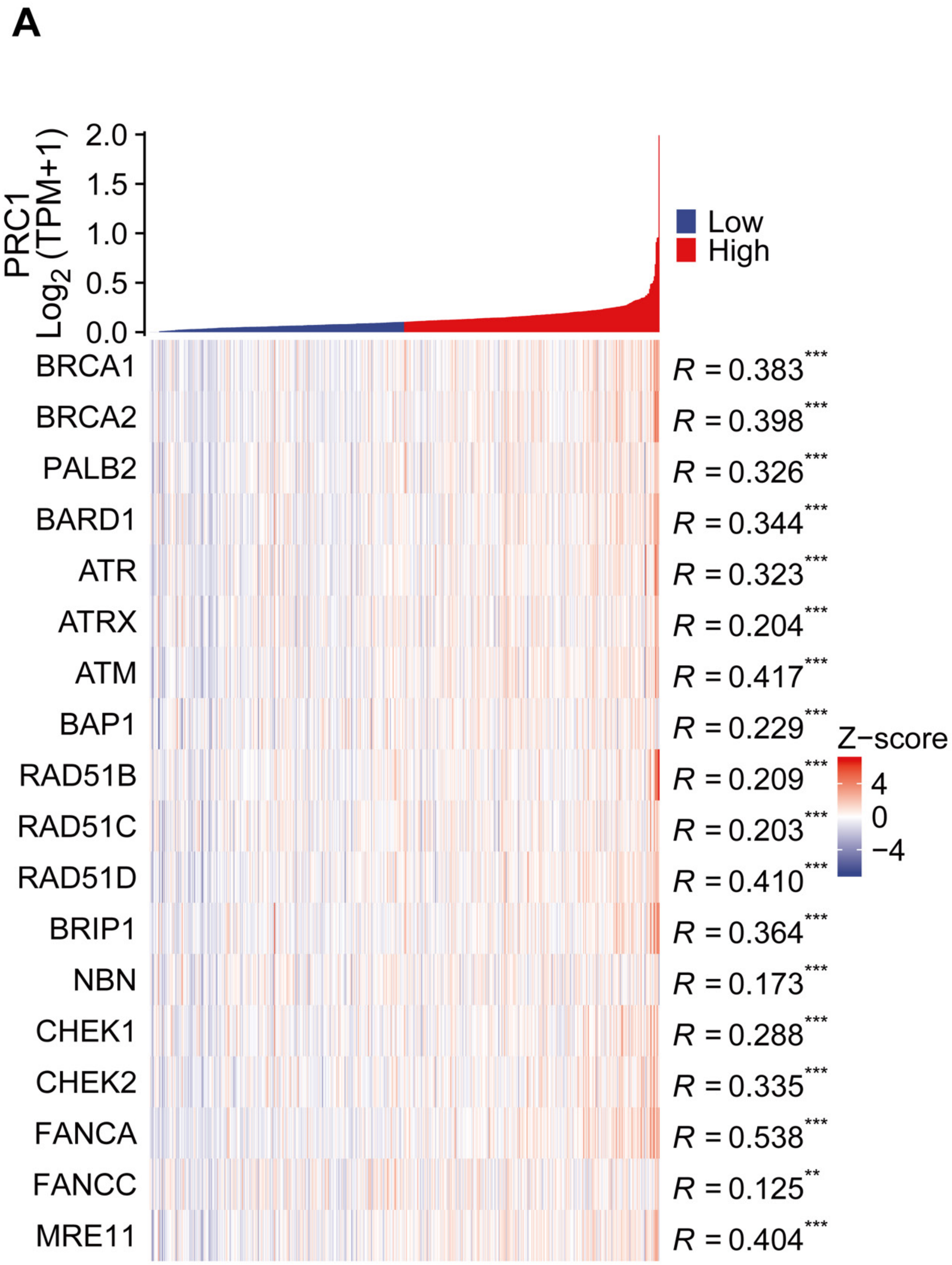

Figure S2. Correlation between PRC1 expression levels and the infiltration of immune cells in the tumor microenvironment. (A). ESTIMATE algorithm. (B). ssGSEA algorithm. (C). Cibersoft algorithm.

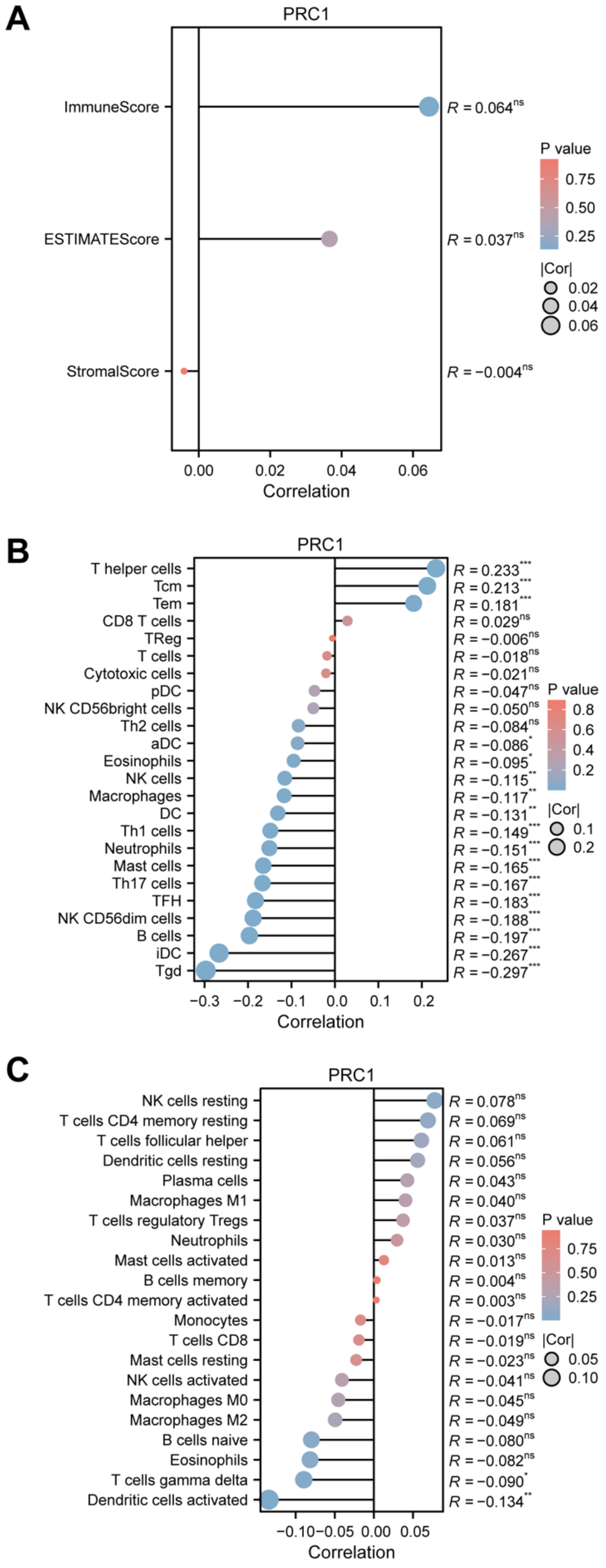

Figure S3. Quantitative bar graphs of in vitro experiments. (A). Quantitative results of EdU assays. (B). Quantitative of Transwell experiments. (C). Quantitative of colony formation assays.

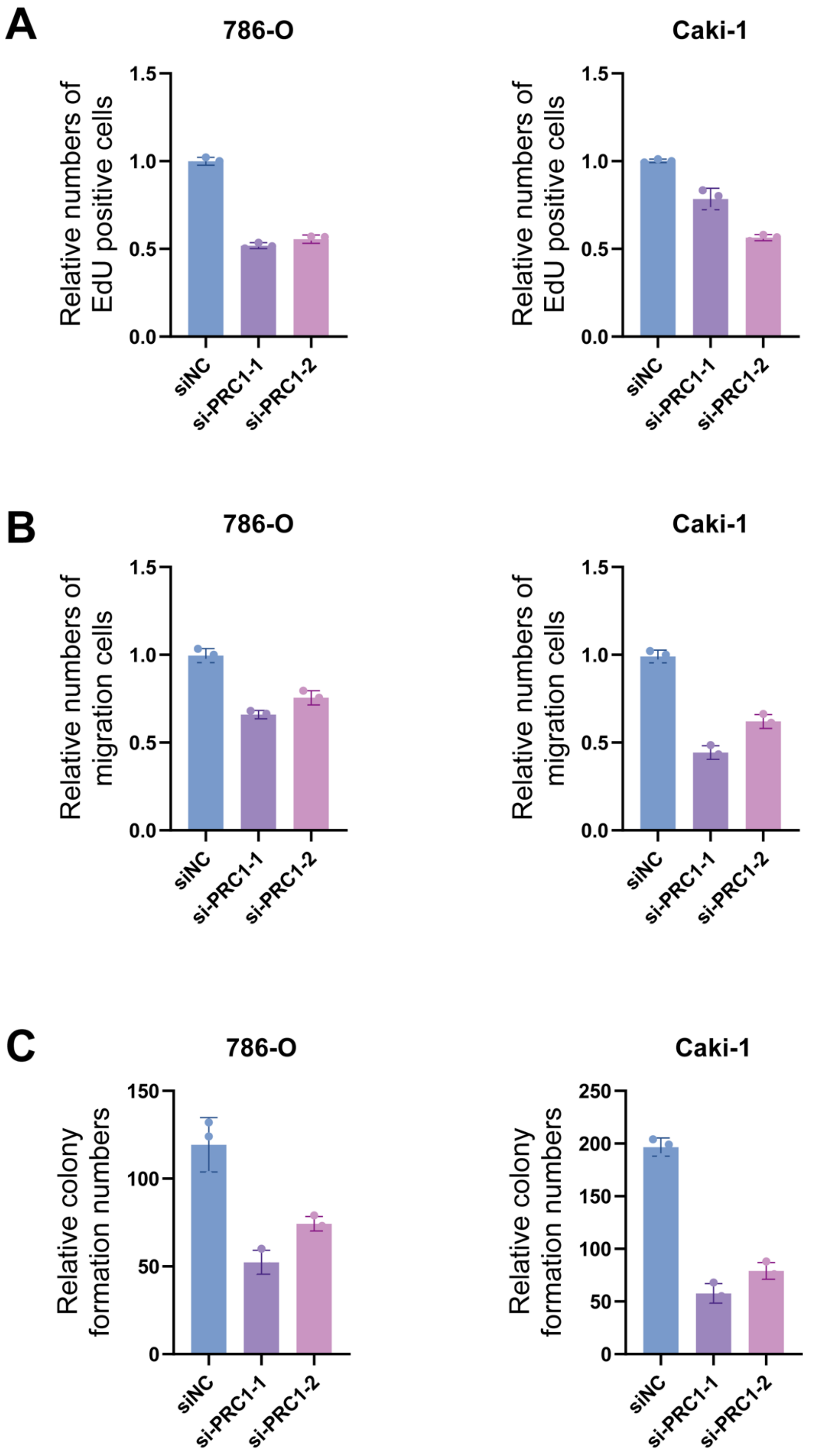

Figure S4. Assessment of the proliferation and migration abilities after knockdown PRC1 in HK-2 cells. (A). CCK-8 assays. (B). Transwell assays.

A

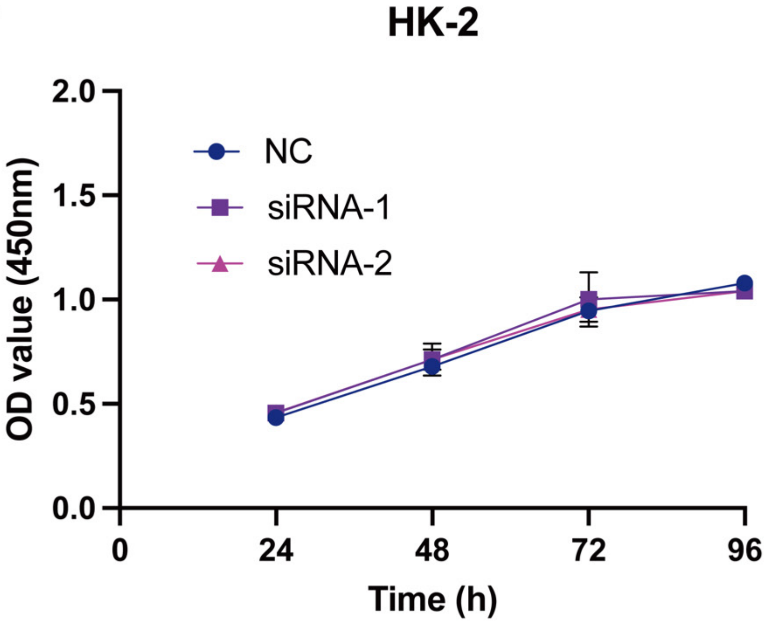

B

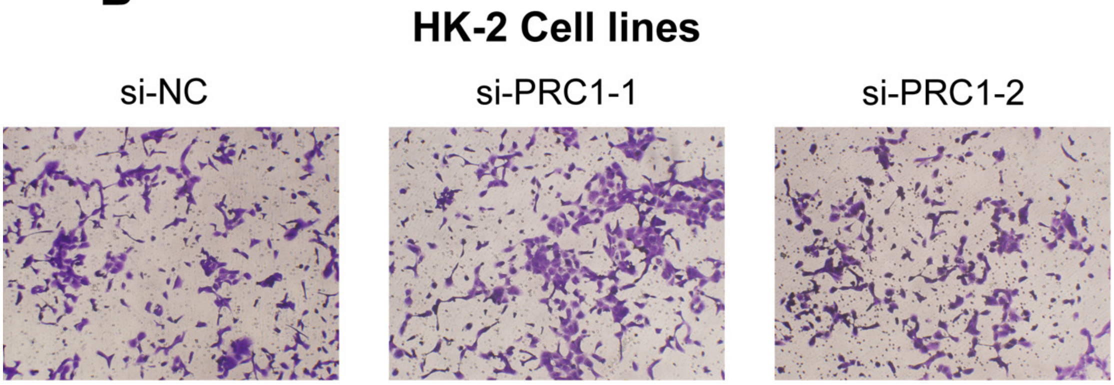

Supplement: Supplementary file 1 — Supplementary figures and tables. [file ijmsv22p1658s1.pdf]
